# Trimming Surface Sugars Protects *Histoplasma* from Immune Attack

**DOI:** 10.1128/mBio.00553-16

**Published:** 2016-04-26

**Authors:** Gordon D. Brown

**Affiliations:** Aberdeen Fungal Group, MRC Centre for Medical Mycology, Institute of Medical Sciences, University of Aberdeen, Foresterhill, Aberdeen, United Kingdom

## Abstract

Dectin-1 is an essential innate immune receptor that recognizes β-glucans in fungal cell walls. Its importance is underscored by the mechanisms that fungal pathogens have evolved to avoid detection by this receptor. One such pathogen is *Histoplasma capsulatum*, and in a recent article in *mBio*, Rappleye’s group presented data showing that yeasts of this organism secrete a β-glucanase, Eng1, which acts to prune β-glucans that are exposed on the fungal cell surface [A. L. Garfoot et al., mBio 7(2):e01388-15, 2016, http://dx.doi.org/10.1128/mBio.01388-15]. The trimming of these sugars reduces immune recognition through Dectin-1 and subsequent inflammatory responses, enhancing the pathogenesis of *H. capsulatum*.

## COMMENTARY

**FIG 1:**
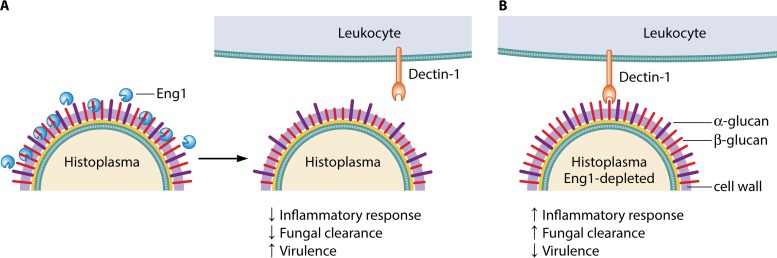
The Eng1 β-glucanase enhances the virulence of *Histoplasma capsulatum* by reducing β-glucan exposure. (A) Upon inhalation by the mammalian host, *H. capsulatum* switches to a yeast morphotype and induces the production of a number of virulence factors. One of these is the β-glucanase Eng1, which is secreted from the yeast and acts to trim off excess β-glucans exposed on the surface of the cell. This helps to prevent recognition by Dectin-1 on leukocytes, resulting in reduced inflammatory responses and increased pathogenesis in infected animals. (B) In strains of *H. capsulatum* where Eng1 has been depleted, Dectin-1-mediated recognition of surface-exposed β-glucan leads to enhanced inflammatory responses and fungal clearance, significantly reducing the virulence of this pathogen.

The immune system detects infection by fungal pathogens through an array of pattern recognition receptors, the most important of which are the C-type lectin receptors (CLRs) (1). Dectin-1 is the best-characterized CLR and recognizes β-glucan, one of the major carbohydrate components of fungal cell walls. Dectin-1 can induce numerous innate and adaptive immune responses and plays an essential role in immunity to several fungal pathogens (2). Its importance is underscored by the mechanisms that fungal pathogens have evolved to avoid detection by this receptor. One such pathogen is *Histoplasma capsulatum*, which causes respiratory and disseminated disease in immunocompetent animals. This organism grows in the environment as a conidium-producing mycelium but switches to a yeast form upon inhalation by the host. In earlier work, Rappleye and colleagues had discovered that the switch to yeast mediates the induction of α-glucan, which overlays the cell wall β-glucan and masks it from Dectin-1-mediated immune recognition (3). In a recent article in *mBio* (4), Rappleye’s group presented new data showing that *H. capsulatum* yeasts also secrete a β-glucanase, Eng1, which acts to prune any residual β-glucans that are exposed on the cell surface. Trimming these sugars reduces immune recognition through Dectin-1 and subsequent inflammatory responses, enhancing the pathogenesis of *H. capsulatum* (Fig. 1).

The switch to yeast morphology induces the expression of many virulence factors, such as α-glucan (described above), in *H. capsulatum*. Rappleye and colleagues had previously characterized the extracellular proteome of yeast cells and had identified a number of proteins that were homologous to glycoside hydrolases, one of which was Eng1 (5). In the current work, the authors demonstrated that the level of transcription of *ENG1* was much higher in yeast cells than in mycelia (4), suggesting that it may be involved in adaptation to life in the mammalian host. To understand the function of this protein, the team generated several strains of *Histoplasma*, in phylogenetically distinct backgrounds, in which the production of Eng1 was substantially reduced using RNA interference (RNAi). They found that depletion of this protein did not affect yeast viability or growth in liquid medium. In other fungi, Eng1 is localized to the cell wall and is required for cell separation during yeast budding (6, 7). Intriguingly, the authors found that the secreted Eng1 did not associate with the cell wall of *Histoplasma* and was not required for cell separation during budding. Given these disparities from other organisms, Rappleye and colleagues then confirmed that Eng1 was a functioning β-glucanase. By measuring enzyme activity against that of the β-glucan, laminarin, they were able to clearly demonstrate that purified Eng1 possessed β-glucanase activity, as did culture filtrates of wild-type *Histoplasma*. Importantly, this activity was significantly reduced in culture filtrates from strains where Eng1 had been depleted using RNAi. Thus, the authors had demonstrated that Eng1 was a functioning β-glucanase but that this enzyme was not required for yeast growth *in vitro*.

Rappleye and colleagues next used murine models to determine if Eng1 was required by *Histoplasma* during infection *in vivo*. Mice infected with Eng1-depleted strains developed significantly reduced fungal burdens in their lungs compared to animals infected with Eng1-sufficient strains. Depletion of Eng1 also led to reduced fungal burdens in the spleen, revealing a role for this enzyme in systemic dissemination. Notably, 2 weeks after infection, mice infected with Eng-1-expressing strains, but not with the Eng1-depleted strains, had become moribund. Thus, Eng1 plays a key role in the virulence of *Histoplasma*.

As a secreted β-glucanase, Rappleye and colleagues postulated that Eng1 may be contributing to virulence by reducing the levels of cell wall β-glucans that would be available for detection by the immune system. To demonstrate this, they made use of a Dectin-1-expressing NIH 3T3 fibroblast cell line and explored the efficiency with which these cells were able to bind the various strains of *Histoplasma*. Indeed, they found that yeast strains depleted of Eng1 bound with 4-fold-greater efficiency to the fibroblasts, showing that more β-glucan was available for recognition by Dectin-1. Indeed, the authors could show that binding to the cell lines was completely β-glucan dependent. Moreover, they found that the increased binding could be reversed following treatment of the Eng1-depleted yeasts with purified Eng1 or with culture filtrates from Eng-1-expressing strains (but not with culture filtrates of Eng1-depleted yeast). However, the effect of Eng-1 depletion on Dectin-1 recognition was not due to gross alterations in the yeast, as the authors found no major changes in the glycan composition or structure of the cell wall. Eng1-depleted yeasts also demonstrated comparable sensitivities to cell wall-destabilizing compounds, detergents, and antifungal drugs (including caspofungin, which targets beta-glucan synthesis). Next, the authors probed the yeast surface with a soluble version of Dectin-1 and visualized binding of this probe by immunofluorescence microscopy. Excitingly, this revealed that the Eng1-depleted strains had substantially increased exposure of β-glucan on their cell surface. These key results revealed that the main function of Eng1 was to prune exposed β-glucans from the yeast cell surface.

Differences in cell wall β-glucan exposure influence inflammatory responses mediated by Dectin-1 and the subsequent development of antifungal immunity (2). To determine if the Eng1-mediated pruning of these carbohydrates could influence leukocyte responses, Rappleye and colleagues examined the production of two inflammatory cytokines (tumor necrosis factor alpha [TNF-α] and interleukin-6 [IL-6]) following exposure of peritoneal macrophages or bone marrow-derived dendritic cells to their various strains of *Histoplasma*. Both leukocyte populations express Dectin-1 (2), and the authors found that depletion of Eng1 led to significantly elevated production of the two cytokines. This response was shown to be Dectin-1 dependent, as it could be inhibited in the presence of a blocking anti-Dectin-1 antibody. Despite the enhanced recognition of the Eng1-depleted strains by Dectin-1, the level of binding to macrophages was not different from that seen with Eng1-producing strains. This is consistent with previous data showing that complement receptors are the major leukocyte receptors involved in the nonopsonic uptake of these yeasts (8). There was also no difference between these strains in viability and intracellular replication in macrophages. This suggests that antimicrobial responses, such as the respiratory burst, induced following activation of Dectin-1 by β-glucans (2) are not responsible for the decreased fungal burdens observed *in vivo*. One caveat with respect to this interpretation pertains to the use of macrophage cell lines in the latter experiments, which often do not fully recapitulate the functionality of primary leukocytes. Crucially, however, the authors demonstrated that the virulence of Eng1-deficient strains was restored upon infection of Dectin-1-deficient mice, providing definitive evidence that the primary function of this glucanase is to reduce host recognition of surface-exposed β-glucans during infection.

In the final set of experiments, Rappleye and colleagues assessed how Eng1 influenced the recognition of *Histoplasma* strains that also use α-glucan to mask exposure of β-glucan. These experiments involved generating yeasts lacking Eng1 or α-glucan or both components and then comparing the levels of exposed β-glucans by measuring the binding of these strains to Dectin-1-expressing NIH 3T3 fibroblasts, as described above. While the loss of Eng1 or α-glucan substantially increased binding, as described previously by the authors (3), the loss of both components led to even higher levels of binding. Thus, the combined role of α-glucan and Eng1 is to minimize β-glucan exposure and hence Dectin-1-mediated immune recognition.

Like all good research, the discoveries reported in this paper raise many issues that require future investigation. One burning issue is why *H. capsulatum* puts so much effort into shielding its β-glucan. This organism is an intracellular pathogen of leukocytes, which possess an array of pattern recognition receptors that can detect other cell wall components. Indeed, Eng1-expressing strains of *H. capsulatum* are sensed by the immune system, as these yeasts still induced inflammatory responses in macrophages and dendritic cells (albeit at lower levels than those induced by Eng1-depleted yeasts). A potential clue lies in the time frame in which Eng1-depleted strains fall under immune control *in vivo*. Eng1-depleted strains show equivalently high fungal burdens early after infection (by day 4, when the innate immune system is primarily responsible for controlling infections) and begin to be cleared only at later time points (after day 8, when adaptive immunity is starting to kick in). This suggests that the effect of Eng1 is to control the influence of Dectin-1 on the development of antifungal adaptive immune responses. In fact, the authors mention that they detected a 3-fold increase in the numbers of IL-17-producing T cells in the lungs of infected mice, an important antifungal adaptive immune response that is induced by Dectin-1 (1). Whatever the underlying mechanism, Rappleye and colleagues have discovered a novel virulence factor that provides important new insights into fungal pathogenesis. Indeed, this report provides evidence that fungal virulence can evolve through reassignment of cell wall machinery that is normally dedicated to distinct functions in other organisms. The question remains whether such mechanisms also exist in other pathogens or if further systems in *Histoplasma* have been similarly coopted for virulence.
